# Drain vs. no-drain for acetabular fractures after treatment *via* a modified stoppa approach: A retrospective study

**DOI:** 10.3389/fsurg.2023.1133744

**Published:** 2023-03-17

**Authors:** Lin Jin, Zhongzheng Wang, Kuo Zhao, Xiaodong Lian, Wei Chen, Yingze Zhang, Zhiyong Hou

**Affiliations:** ^1^ Department of Orthopaedic Surgery, Third Hospital of Hebei Medical University, Shijiazhuang, China; ^2^ Key Laboratory of Biomechanics of Hebei Province, Third Hospital of Hebei Medical University, Shijiazhuang, China; ^3^ NHC Key Laboratory of Intelligent Orthopaedic Equipment, Third Hospital of Hebei Medical University, Shijiazhuang, China; ^4^ Chinese Academy of Engineering, Beijing, China

**Keywords:** acetabular fracture, close suction drainage, modified stoppa approach, surgical method, treatment

## Abstract

**Objective:**

The aim of this study was to compare the clinical efficacy of close suction drainage (CSD) and no-CSD after a modified Stoppa approach for the surgical fixation of acetabular fractures.

**Methods:**

This retrospective study included 49 consecutive acetabular fracture patients, who presented to a single level I trauma center for surgical fixation, using a modified Stoppa approach from January 2018 to January 2021. All surgeries were performed by a senior surgeon using the same approach, and the patients were divided into two groups based on whether CSD was used after the operation. Details of the patient demographics, fracture characteristics, intraoperative indicators, reduction quality, intra and postoperative blood transfusion, clinical outcomes, and incision-related complications were collected.

**Results:**

No significant differences were found in the demographics, fracture characteristics, intraoperative indicators, reduction quality, clinical outcomes, and incision-related complications between the two groups (*P *> 0.05). The use of CSD was associated with a significantly higher postoperative blood transfusion volume (*P = *0.034) and postoperative blood transfusion rate (*P = *0.027). In addition, there was a significant difference in postoperative temperatures, especially on postoperative Day 2 (no-CSD 36.97 ± 0.51°C vs. CSD 37.34 ± 0.69°C, *P = *0.035), and higher visual analogue scale (VAS) scores, especially on postoperative Day 1 (no-CSD 3.00 ± 0.93 vs. CSD 4.14 ± 1.43, *P = *0.002) and 3 (no-CSD 1.73 ± 0.94 vs. CSD 2.48 ± 1.08, *P = *0.013).

**Conclusion:**

The results of this study suggest that routine use of CSD should not be recommended for patients with acetabular fractures after surgical fixation using a modified Stoppa approach.

## 1. Introduction

Acetabular fractures are usually caused by high-energy trauma, have an incidence of ∼3 out of every 100,000 patients per year, and are one of the most difficult fractures to manage in orthopedic surgery (1, 2). Because it is an intraarticular fracture, displaced acetabular fracture patients usually need surgical treatment (3). Many surgical approaches have been used to treat acetabular fractures, such as the iliofemoral, ilioinguinal, Kocher–Langenbeck, combined anterior/posterior, modified Stoppa, and extended iliofemoral approaches (4–6). The modified Stoppa approach was proposed by Cole and Bolhofner in 1994 and has been widely used in recent years (7). It is an ideal surgical fixation method for the majority of acetabular fractures, because it not only results in good exposure of the fracture area but also achieves a good reduction of fracture fragments, especially those involving the anterior column, anterior wall, and posterior hemitransverse, transverse, T-type, and both columns with predominately anterior displacement (2, 4, 8, 9).

In clinical applications, closed suction drainage (CSD) is often used as a preventive strategy to eliminate dead space and reduce the occurrence of postoperative wound complications, including hematoma formation, redness, infection, pain, and incisional hernia (10, 11). The application of CSD in open wound or infectious surgeries can play a role in draining pus and necrotic tissue debris, and its use in these cases was previously beyond doubt (11). However, there is little published evidence to support its routine use after surgery. Some literature suggests that the use of CSD may be associated with increased postoperative blood transfusion rates, days in hospital, and incision pain (10, 12). In addition, some have argued that CSD, as a foreign body, may become a potential source of infection, allowing external pathogens to enter deep wounds and increasing the chances of infection (13–15). Similar debates have been reported in the orthopedic spine and trauma literature (16, 17).

To prevent the formation of wound hematomas and enhance postoperative recovery, many orthopedic surgeons still routinely use CSD after using a modified Stoppa approach to treat acetabular fractures. However, we have found that the use of CSD will not provide any benefit to patients with acetabular fractures repaired using a modified Stoppa approach. Therefore, the aim of this study was to compare the clinical efficacy of CSD with no-CSD after a modified Stoppa approach in the surgical fixation of acetabular fractures.

## 2. Patients and methods

### 2.1. Patients

**Figure 1:**
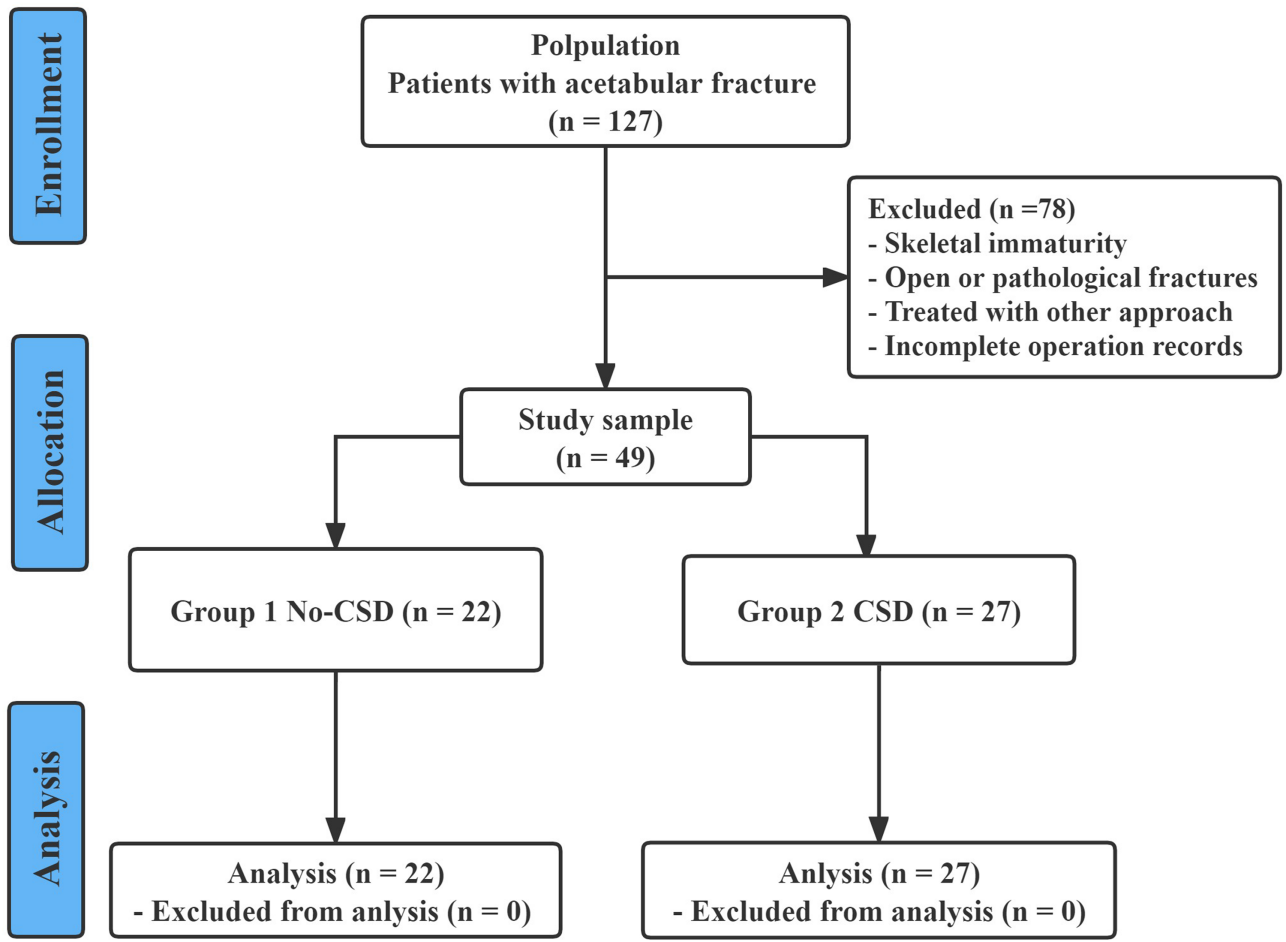
The flowchart of the patient screening process.

Between January 2018 and January 2021, a total of 127 skeletally mature patients with acetabular fractures were surgically treated in our level I trauma center. The inclusion criteria included the following: (1) adult patients aged 18 years or older; (2) diagnosis of an acute and closed acetabular fracture; (3) patients who underwent open reduction and internal fixation *via* a modified Stoppa approach; and (4) complete and available imaging and hospitalization data. The exclusion criteria were as follows: (1) open acetabular fractures; (2) previous history of acetabular fracture or bone tumor; (3) patients who underwent surgical treatment *via* another approach; and (4) patients with incomplete hospitalization and operation records. A total of 49 patients with acetabular fractures who underwent surgical treatment through a modified Stoppa approach were enrolled in our study for analysis. All 49 patients were divided into two groups according to whether CSD was used after surgery (in Group 1, 22 patients underwent no-CSD after surgery; in Group 2, 27 patients underwent CSD after surgery). Figure 1 shows a chart of the study design.

This study was approved by the Ethics Committee of Third Hospital of Hebei Medical University (Theoretical No. K2015-001-12) and conformed to the ethical standards of the Declaration of Helsinki, adopted in 1964, and its subsequent amendments. Signed informed consent was obtained from all patients.

### 2.2. Preoperative management

All patients were hemodynamically stable before the operation. When available, radiography and CT scans of the injured hip were performed in all patients to assess the extent of displacement. According to the imaging data results, these fractures were graded by the Judet–Letournel classification system (18). If no contraindication was present, low-molecular-weight heparin was given to the patients before the operation. For unstable fractures, bone traction or skin traction was used to facilitate intraoperative reduction.

### 2.3. Surgical techniques

**Figure 2:**
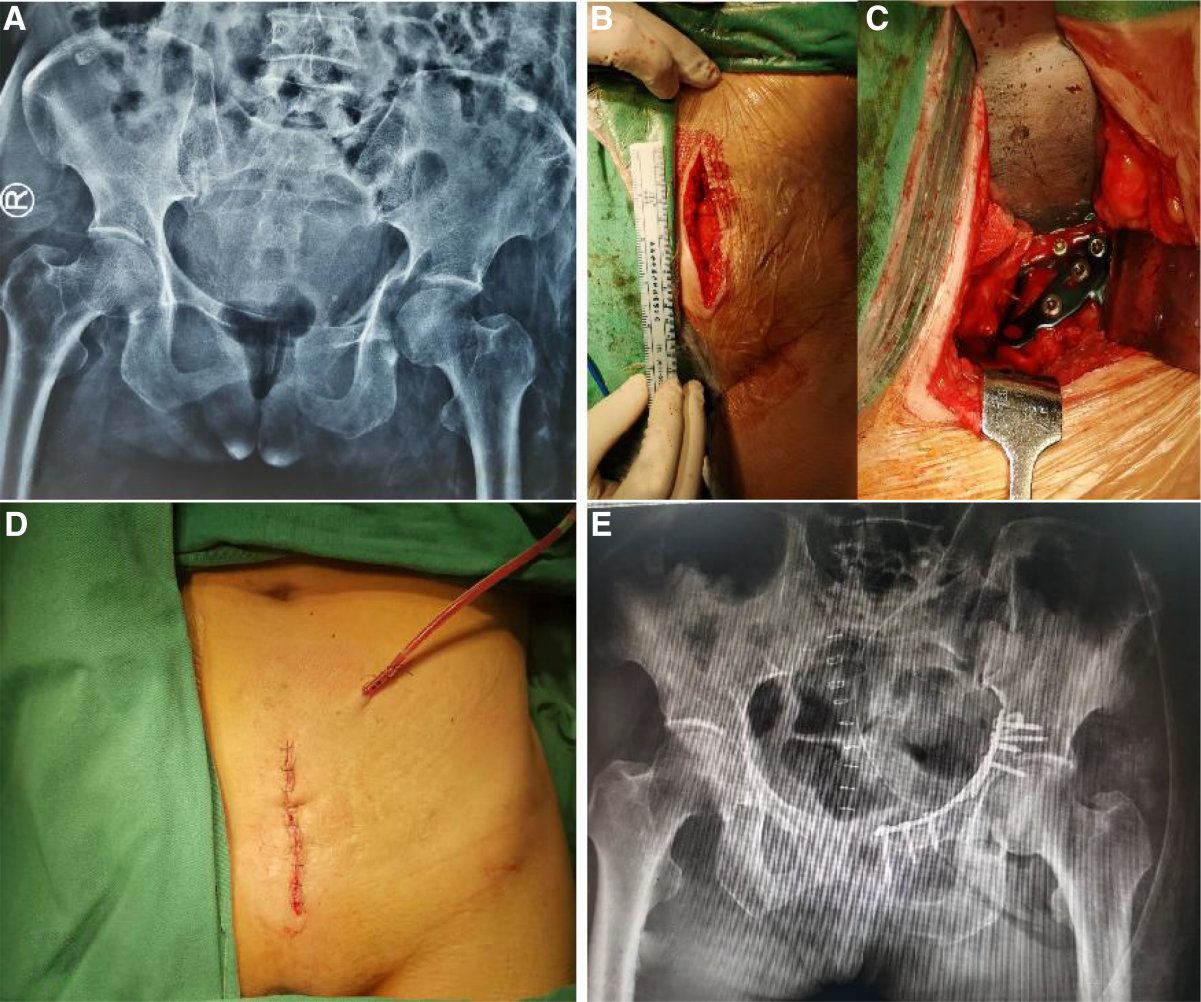
A female patient, 57 years old, with a T-type acetabular fracture who underwent surgical treatment *via* a modified stoppa approach. (**A**) preoperative anteroposterior pelvic radiograph; (**B**), picture of the incision of the modified Stoppa approach; (**C**), internal fixation was placed after fracture reduction; (**D**), a CSD was used after surgery; (**E**), postoperative anteroposterior pelvic radiograph.

All patients underwent general anesthesia in the supine position during the operation. A vertical midline incision was made from approximately 2 cm below the umbilicus to 1 cm above the pubic symphysis joint. After making an incision along the alba line of the rectus abdominis muscle, blunt finger dissection was performed to detach the superior ramus of the pubis, releasing the periosteum and iliopectineal fascia, extending to the pelvic brim and the internal iliac fossa, and continuing to emerge laterally. During exposure, the anastomotic branches (corona mortis) between the internal and external iliac vessels were identified for protection. Tying or cauterization was performed to avoid bleeding if the corona mortis was damaged. Subperiosteal dissection was performed along the pelvic brim, exposing the fracture fragments. Attention was paid to identify and protect the obturator nerves and blood vessels during the further operation. Once the fracture site was exposed, reduction tools were used to attempt reduction and internal fixation. After confirmation by fluoroscopy, the wounds were closed in layers for the patients in Group 1. For the patients in Group 2, all surgical procedures were same as those in Group 1, except that a drain for CSD was placed before closing the wound (Figure 2).

### 2.4. Postoperative care

Patients in both groups received the same postoperative rehabilitation program. Low-molecular-weight sodium heparin was given to prevent deep vein thrombosis from 6 h to 4 weeks after surgery. The drain CSD was removed 48 h after surgery if the drainage did not exceed 50 ml/day. After surgery, the patients were encouraged to perform passive hip mobility as soon as possible. According to the general condition of the patient, the severity of the injury, and the quality of the reduction and fixation, the patients were guided to perform gradual rehabilitation exercises.

### 2.5. Observation index

In the present study, demographic characteristics, fracture characteristics, and medical records, including operative reports and inpatient progress notes, were collected retrospectively and compared between the two groups. The primary outcome measures were perioperative hemoglobin changes, postoperative blood transfusion volume and rate, and postoperative clinical outcomes. The secondary outcome measures included changes in body temperature from before surgery and on postoperative Day 1, 2, 3, and 7, as well as incision-related complications. Data on the duration of operation, intraoperative blood loss, and corona mortis ligation were obtained from the operation records. Data on the preoperative hemoglobin (Hgb) and intra and postoperative blood transfusion volume and rate were obtained from the inpatient progress notes. *Δ*Hgb was expressed as the Hgb change from immediately before to after surgery. The Harris hip score (HHS) was used to evaluate the function of the hip after surgery. Visual analogue scale (VAS) scores were used to evaluate the degree of pain before surgery and on postoperative Day 1, 3, and 7. Incision-related complications mainly included wound infections, wound dehiscence, hematomas, and non-union. Wound-related complications and other perioperative issues were followed up for at least one year.

### 2.6. Statistical analysis

The analyses were performed using SPSS 23.0 software (IBM, Armonk, New York). Measurement data were expressed as the mean ± SD or numbers and percentages (%). The Kolmogorov–Smirnov test was used to confirm a normal distribution. Potential explanatory variables were evaluated using a chi-square test or Fisher's test for categorical variables and independent-samples *t* tests or Mann–Whitney U tests for continuous variables. The level of significance was set at *P* < 0.05.

## 3. Results

### 3.1. Patient and fracture characteristics

In the present study, 49 patients with acetabular fractures who met our screening criteria were enrolled in the analysis, of whom 37 were male and 12 were female (mean age: 49.06 ± 15.28; range: 21–80 years). Among them, 22 patients (44.9%) did not use CSD after surgery *via* a modified Stoppa approach and were enrolled in Group 1. According to the Judet–Letournel classification, there were six fracture types managed by a single modified Stoppa approach, including column fractures (16/49, 32.7%), anterior column fractures (12/49, 24.5%), anterior wall fractures (3/49, 6.1%), anterior column and posterior hemitransverse fractures (5/49, 10.2%), transverse fractures (7/49, 14.3%), and T-type fractures (6/49, 12.2%). All fracture types were identified by the same senior orthopedic surgeon according to the preoperative radiographs and CT scans. There were no significant differences in demographics or fracture classifications between the two groups (Table 1).

**Table 1:** Patient demographic data and fracture characteristics (*n* = 49).

Variable	No-drain Group (*n* = 22)	Drain Group (*n* = 27)	*P* value
Mean age (mean ± SD, years)	18.82 ± 17.19	49.26 ± 13.86	0.921
Sex (male), *n* (%)	17 (77.27)	20 (74.07)	0.796
BMI (mean ± SD, Kg/m^2^)	25.20 ± 2.63	25.87 ± 3.31	0.441
Tobacco smoker (yes), *n* (%)	4 (18.18)	7 (25.93)	0.518
Alcohol use (yes), *n* (%)	3 (13.64)	5 (18.52)	0.646
**Comorbidities (yes), *n* (%)**
Diabetes mellitus	5 (22.73)	3 (11.11)	0.274
Hypertension	6 (27.27)	7 (25.93)	0.915
Coronary heart disease	2 (0.90)	3 (11.11)	0.816
Affected side (left), *n* (%)			0.757
Left	9 (40.91)	13 (48.15)	
Right	12 (54.55)	12 (44.44)	
Bilateral	1 (4.54)	2 (7.41)	
Injury to surgery (mean ± SD, days)	5.00 ± 2.51	5.18 ± 2.82	0.762
Days in hospital (mean ± SD, days)	16.09 ± 3.89	16.48 ± 4.73	0.757
Follow-up (mean ± SD, months)	26.91 ± 17.20	26.22 ± 16.31	0.840
Fracture classification (Judet–Letournel), *n* (%)			0.947
Both columns	8 (36.36)	8 (29.64)	
Anterior column	5 (22.73)	7 (25.93)	
Anterior wall	2 (9.09)	1 (3.70)	
Anterior column and posterior hemitransverse	2 (9.09)	3 (11.11)	
Transverse	3 (13.64)	4 (14.81)	
T-type	2 (9.09)	4 (14.81)	

### 3.2. Perioperative-related indicators

Perioperative variables are shown in Table 2. There were no significant differences observed in intraoperative indicators between the two groups, including the duration of operation, intraoperative blood loss, reduction quality, corona mortis ligation, preoperative Hgb, changes in Hgb from immediately before to after surgery (*Δ*Hgb), and intraoperative blood transfusion volume and rate. While the postoperative blood transfusion volume and rate tended to be higher in the CSD group than in the no-CSD group, the differences were statistically significant (*P *= 0.034 and *P *= 0.027, respectively). There were no significant intergroup differences in incision exudation time (*P *= 0.789) or HHS (*P *= 0.869). Preoperatively, no significant differences were found in body temperature or VAS pain score between the two groups. Postoperatively, there were significant intergroup differences in body temperature on postoperative Day 2 (no-CSD group, 36.97 ± 0.51°C vs. CSD group, 37.34 ± 0.69°C; *P *= 0.035) and VAS pain score on postoperative Day 1 (no-CSD group, 3.00 ± 0.93 vs. CSD group,4.14 ± 1.43; *P *= 0.002) and 3 (no-CSD group,1.73 ± 0.94 vs. CSD group, 2.48 ± 1.08; *P *= 0.013).

**Table 2:** Comparison of perioperative-related indicators between the two groups.

Variable	No-drain Group (*n* = 22)	Drain Group (*n* = 27)	*P* value
ASA score, *n* (%)			0.639
I	2 (9.09)	5 (18.52)	
II	14 (63.64)	15 (55.55)	
III or above	6 (27.27)	7 (25.93)	
Duration of operation (minutes), *n* (%)			0.518
1–120	4 (18.18)	7 (25.93)	
>120	18 (82.82)	20 (74.07)	
Intraoperative blood loss (ml), *n* (%)			0.612
1–200	3 (13.64)	6 (22.22)	
201–400	10 (45.45)	9 (33.33)	
>400	9 (40.91)	12 (44.45)	
Reduction quality, *n* (%)			0.983
Excellent	4 (18.18)	5 (18.52)	
Good	11 (50.00)	14 (51.84)	
Fair	5 (22.73)	5 (18.52)	
Poor	2 (9.09)	3 (11.11)	
Crown of death ligate, *n* (%)	6 (27.27)	6 (22.22)	0.683
Preoperative Hgb (mean ± SD, g/L)	117.28 ± 15.87	112.68 ± 15.06	0.305
*Δ*Hgb (mean ± SD, g/L)	21.34 ± 11.15	17.31 ± 12.46	0.244
Intraoperative blood transfusion volume (mean ± SD, ml)	284.54 ± 200.73	303.70 ± 228.41	0.723
Intraoperative blood transfusion rate, *n* (%)	14 (63.64)	16 (59.26)	0.754
Postoperative blood transfusion volume (mean ± SD, ml)	127.27 ± 169.54	259.25 ± 246.92	0.034
Postoperative blood transfusion rate, *n* (%)	6 (27.27)	15 (55.55)	0.027
Postoperative drainage volume (mean ± SD, ml)	–	153.70 ± 107.56	–
Incision exudation time (mean ± SD, days)	3.27 ± 1.35	3.37 ± 1.18	0.789
HHS (mean ± SD)	83.56 ± 9.81	84.41 ± 6.61	0.869
**Body temperature (mean ± SD, °C)**
Preoperative	36.82 ± 0.43	36.91 ± 0.40	0.460
Postoperative day 1	37.01 ± 0.55	37.26 ± 0.48	0.107
Postoperative day 2	36.97 ± 0.51	37.34 ± 0.69	0.035
Postoperative day 3	36.92 ± 0.58	37.12 ± 0.41	0.234
Postoperative day 7	36.75 ± 0.33	36.66 ± 0.31	0.488
**VAS pain score (mean ± SD)**
Preoperative	3.77 ± 1.48	3.93 ± 1.66	0.738
Postoperative day 1	3.00 ± 0.93	4.14 ± 1.43	0.002
Postoperative day 3	1.73 ± 0.94	2.48 ± 1.08	0.013
Postoperative day 7	1.04 ± 0.95	1.48 ± 1.01	0.117
Time to remove stitches (mean ± SD, days)	14.41 ± 0.89	14.07 ± 0.96	0.832

### 3.3. Postoperative incision-related complications

There were no significant differences in postoperative incision-related complications between the two groups (*P *> 0.05). Three patients (3/22) in the no-CSD group and five patients (5/27) in the CSD group suffered superficial wound infections that were completely improved by effective antibiotics. Two patients (2/22) in the no-CSD group and three patients (3/27) in the CSD group developed a hematoma, which was absorbed after one week of local physiotherapy. All specific data on incision-related complications are summarized in Table 3.

**Table 3:** Detailed presentation of postoperative incision-related complications between the two groups.

Variable	No-drain Group (*n* = 22)	Drain Group (*n* = 27)	*P* value
**Wound infections, *n* (%)**
Superficial infections	3 (13.64)	5 (18.52)	0.715
Deep infections	0 (0.00)	0 (0.00)	1.000
Wound dehiscence, *n* (%)	0 (0.00)	0 (0.00)	1.000
Hematoma, *n* (%)	2 (9.09)	3 (11.11)	0.816
Nonunion, *n* (%)	0 (0.00)	0 (0.00)	1.000

## 4. Discussion

To our knowledge, our study is the first to evaluate whether using CSD has an impact on the clinical outcome of acetabular fractures treated by a modified Stoppa approach. Previous studies have suggested that CSD could be used as a preventive treatment to decrease the likelihood of surgical site infection and incision-related complications in high-risk patients after orthopedic surgery (10, 19). However, according to our study results, compared to the patients in the no-CSD group, the patients with CSD for acetabular fractures repaired by a modified Stoppa approach were associated with a higher risk of postoperative blood transfusion and pain, with no impact on clinical outcomes and incision-related complications.

Acetabular fracture is one of the most difficult fractures to manage in orthopedic surgery. An ideal surgical approach for acetabular fractures is invaluable. It not only facilitates good exposure of the surgical field and effective reduction of the fracture but also causes as few complications as possible (2). The modified Stoppa approach is only appropriate in specific surgical indications and does not often allow for posterior column access if needed. This approach has many advantages, including large areas of visualization, less chance of damaging major nerves and vessels, short operation time, and less trauma (4, 20).

Traditionally, CSD has been successfully used in the management of acute and chronic open wounds to decrease the incidence of hematomas and infection by reducing the accumulation of blood and exudate in the incision cavity (10, 21, 22). In recent years, researchers have tried to apply CSD to closed incisions for acetabular fractures to prevent surgical site infection and wound complications after surgery (19). A retrospective study by Reddix et al. (23) showed that the use of CSD in patients with acetabular fracture may significantly reduce the incidence of deep infections. Although certain high-risk patients with acetabular fractures, such as obese patients, may benefit from treatment with CSD, the use of CSD remains controversial. For example, the results published by Boissonneault et al. (24) show that, in their study, the use of CSD after the surgical fixation of acetabular fractures *via* the Kocher–Langenbeck approach significantly increased postoperative blood transfusion rates and days in hospital, but wound complications and incision infection rates did not differ.

Similarly, in the current study, the patients in the CSD group showed higher postoperative transfusion volumes (259.25 ml vs. 127.27 ml, *P* = 0.034) and transfusion rates (55.55% vs. 27.27%, *P* = 0.027) than those in the no-CSD group. Some scholars believe that 24–48 h after surgery is the stage of maximum bleeding and seepage of surgical sites (25). Negative pressure suction caused the exudate to continue to drain outside the body and disrupted the self-coagulation mechanism, which were the main reasons for the increase in postoperative blood transfusion (25). In fact, prophylactic CSD after acetabular fractures remains controversial. The use of CSD is almost always determined by the surgeon after surgery based on the quality of intraoperative hemostasis, magnitude of the surgical procedure, and experience (17, 26).

Several authors have reported that the advantages of using CSD after acetabular fracture surgery were controlling postoperative wound drainage and preventing local accumulation of hematomas (27). However, Kim et al. (28) found that routine use of CSD not only failed to prevent or reduce postoperative morbidities but also may have led to prolonged postoperative pain. Some published studies confirmed that the application of CSD may produce undesired results, such as an increased risk of infection and pain and an increased length of hospital stay after surgery. In addition, some studies suggested that compared with the CSD group, the VAS scores of the patients in the no-CSD group decreased by 50%, especially on postoperative Day 2 (26).

In our study, we found that there was no increase in the frequency of incision-related complications in the no-CSD group. A total of three patients (11.11%) developed hematomas and five (18.52%) developed superficial infections in the CSD group, which were no significant difference from those in the no-CSD group. Theoretically, the use of CSD is beneficial to reduce the incidence of pain and fever. However, our findings were the opposite, with the patients in the CSD group having higher VAS scores on postoperative Day 1 (CSD group, 4.14 ± 1.43 vs. no-CSD group, 3.00 ± 0.93; *P *= 0.002) and 3 (CSD group, 2.48 ± 1.08 vs. no-CSD group, 1.73 ± 0.94; *P *= 0.013) and higher temperatures on postoperative Day 2 (CSD group, 37.34 ± 0.69°C vs. no-CSD group, 36.97 ± 0.51°C; *P *= 0.035) than the patients in the no-CSD group. We believed that the drain itself, similar to a foreign body, increased discomfort and anxiety. In addition, active pulling and removal can also increase patient pain, and the absorption of residual exudate in the cavity after removal of the drain can cause fever.

This study has several potential limitations, including the retrospective design with the associated bias. The small sample size from a single institution was also a main limitation. In addition, the retrospective evaluation of intra and postoperative blood loss from medical records may lead to concerns about the reliability of this study. We only compared and analyzed a few perioperative laboratory indicators, while other indicators, such as white blood cell count and platelet count, were not addressed. Furthermore, we only compared the placement of drains after treating acetabular fractures *via* a modified Stoppa approach and did not include other approaches or the number of drains. Finally, although it is an important evaluation indicator in the perioperative period of hip fracture, the incidence of deep vein thrombosis was not included due to incomplete data. We will conduct further research in the future.

## 5. Conclusions

It is unnecessary to use CSD routinely after treating acetabular fractures *via* a modified Stoppa approach, because the application of CSD will not effectively reduce the incidence of postoperative incision-related complications and will increase the risk of increased postoperative blood transfusion rate and pain.

## Data Availability

The original contributions presented in the study are included in the article/Supplementary Material, further inquiries can be directed to the corresponding author/s.
